# EF-hand domain containing 2 (Efhc2) is crucial for distal segmentation of pronephros in zebrafish

**DOI:** 10.1186/s13578-018-0253-z

**Published:** 2018-10-16

**Authors:** Praveen Barrodia, Chinmoy Patra, Rajeeb K. Swain

**Affiliations:** 1 Institute of Life Sciences, Nalco Square, Chandrasekharpur, Bhubaneswar, Odisha 751023 India; 2 Manipal Academy of Higher Education, Manipal, Karnataka 576104 India; 3 Agharkar Research Institute, Pune, Maharashtra India; 4 Savitribai Phule Pune University, Pune, Maharashtra India

**Keywords:** Pronephros, Retinoic acid, Multi-ciliated cells, Efhc2, Zebrafish

## Abstract

**Background:**

The blood filtering organ in zebrafish embryos is the pronephros, which consists of two functional nephrons. Segmentation of a nephron into different domains is essential for its function and is well conserved among vertebrates. Zebrafish has been extensively used as a model to understand nephron segmentation during development. Here, we have identified EF-hand domain containing 2 (Efhc2) as a novel component of genetic programme regulating nephron segmentation in zebrafish. Human EFHC2 is a protein with one predicted calcium-binding EF-hand motif and three DM10 domains, whose function is unknown. EFHC2 has been implicated in several brain-related genetic diseases like Turner syndrome and juvenile myoclonic epilepsy. However, there is limited information on its normal physiological function.

**Results:**

*efhc2* mRNA is primarily expressed in the pronephros of zebrafish embryos. Other sites of expression include olfactory placode, notochord, otic vesicle, epiphysis and neuromast cells. Morpholino antisense oligonucleotide-mediated knock-down of Efhc2 resulted in defects in pronephros development and function in zebrafish embryos. Efhc2 knock-down leads to expansion of distal early segment of pronephros, whereas, the corpuscle of stannius and distal late segments were reduced. The number of multi-ciliated cells (MCC) that are present in a salt-and-pepper fashion throughout the middle of each nephron and vital for fluid flow were also reduced. It is known that retinoic acid (RA) signaling regulates pronephros segmentation in vertebrates and we show that Efhc2 function is crucial for nephron segmentation in zebrafish. Our data suggests that RA and Efhc2 function independent of each other in pronephros segmentation. However, Efhc2 and RA synergistically regulate MCC development.

**Conclusion:**

In this study, we have identified Efhc2 as a regulator of segmentation of the distal part of nephron and pronephros function during zebrafish development.

**Electronic supplementary material:**

The online version of this article (10.1186/s13578-018-0253-z) contains supplementary material, which is available to authorized users.

## Background

The vertebrate kidney is an excretory organ that arises from the intermediate mesoderm during embryogenesis [1]. The functional and structural unit of a kidney is the nephron [2]. Kidney maintains the blood plasma by filtration, reabsorption, secretion and excretion. Based on the complexity of nephron, kidneys are classified into three categories; pronephros, mesonephros and metanephros. In mammals, pronephros is vestigial, mesonephros is embryonic kidney and metanephros is adult kidney [3]. In fishes and frogs, pronephros serves as an embryonic excretory organ [4]. Zebrafish pronephros is a simple organ made up of two nephrons originating from intermediate mesoderm [5]. These two nephrons share a common glomerulus at the anterior end and are joined together by a single cloaca at the posterior end. By 24 hpf, zebrafish pronephros can be divided into eight distinct segments that have strong resemblance to nephron segments of other vertebrates including humans [6]. These segments can be visualized by the expression of genes in specific segments. The glomerulus (G) (expresses *wt1a*, *wt1b*, *mafb*), neck (N) (expresses *pax2a*, *cdh17*, *NBC1*), proximal convoluted tubule (PCT) (expresses *slc20a1a*, *slc26a2*, *pdzk1*), proximal straight tubule (PST) (expresses *trpm7*, *slc26a2*, *pdzk1*), distal early (DE) (expresses *slc12a1*, *ROMK2*), corpuscle of stannius (CS) (expresses *stc1*, *sall1*), distal late (DL) (expresses *slc12a3*, *evil*, *ret1*), and pronephric duct (PD) (expresses *gata3*, *evi1*, *ret1*) [7]. Kidney organogenesis in zebrafish is under the control of several transcription factors (TFs) and signaling pathways [8]. These regulatory molecules are important for specification and proper segmentation of pronephros in zebrafish. Transcription factors *pax2a* and *pax8* are reported to be important in specification of pronephros in vertebrates [9]. Zebrafish pronephros can be divided into two territories; proximal and distal. The development of most anterior part, the podocyte is regulated by *wt1a* and notch signaling components *deltaC*, *jagged1b*, *jagged2a*, *rbpJ*, and *hey1* [10]. Proximal segments of the pronephros are under the control of *pax2a*, *pax8* and *jagged2b* genes [10]. Transcriptional factor *irx3b*, *evi1*, and *pou3f3a*/*pou3f3b* are required for the development of distal segments of pronephros [6]. The *hnf1ba* and *hnf1bb* are required for proper nephron patterning by regulating the expression of other genes, like *pax2a* establishes boundary of podocyte and the neck by directly inhibiting *wt* mediated podocyte formation [11]. Knock-down of *hnf1ba* and *hnf1bb* showed defects in the formation of proximal and distal segments of pronephros, indicating their role in nephron segmentation [12, 13]. Caudal type homeobox (*cdx*) transcription factors such as *cdx4* and *cdx1a* have been shown to regulate position of pronephros along the anterior–posterior (A–P) axis [7]. The transcription factor *mecom* and *sim1a* are required for formation of the distal tubule and restriction of proximal segments of the nephron [14].

The role of retinoic acid (RA) signaling in the proximal–distal segmentation of pronephros is well explored during zebrafish embryogenesis [7, 15]. Zebrafish embryos deficient in RA synthesis show expanded distal segments, whereas the proximal segments are either reduced or completely absent. Perturbation of retinoic acid signaling results in severe defects in nephron segmentation and pronephros function [6, 7]. Conversely, exogenous treatment of RA leads to the formation of pronephros with expanded proximal segments and reduced distal segments. These results indicate that RA promotes proximal segmentation and limits the formation of distal segments [7]. In this study, we show that Efhc2 is required for the proper segmentation of the distal parts of pronephros and development of multi-ciliated cells (MCC).

## Results

### Expression pattern of *efhc2* in zebrafish embryos

**Fig. 1:**
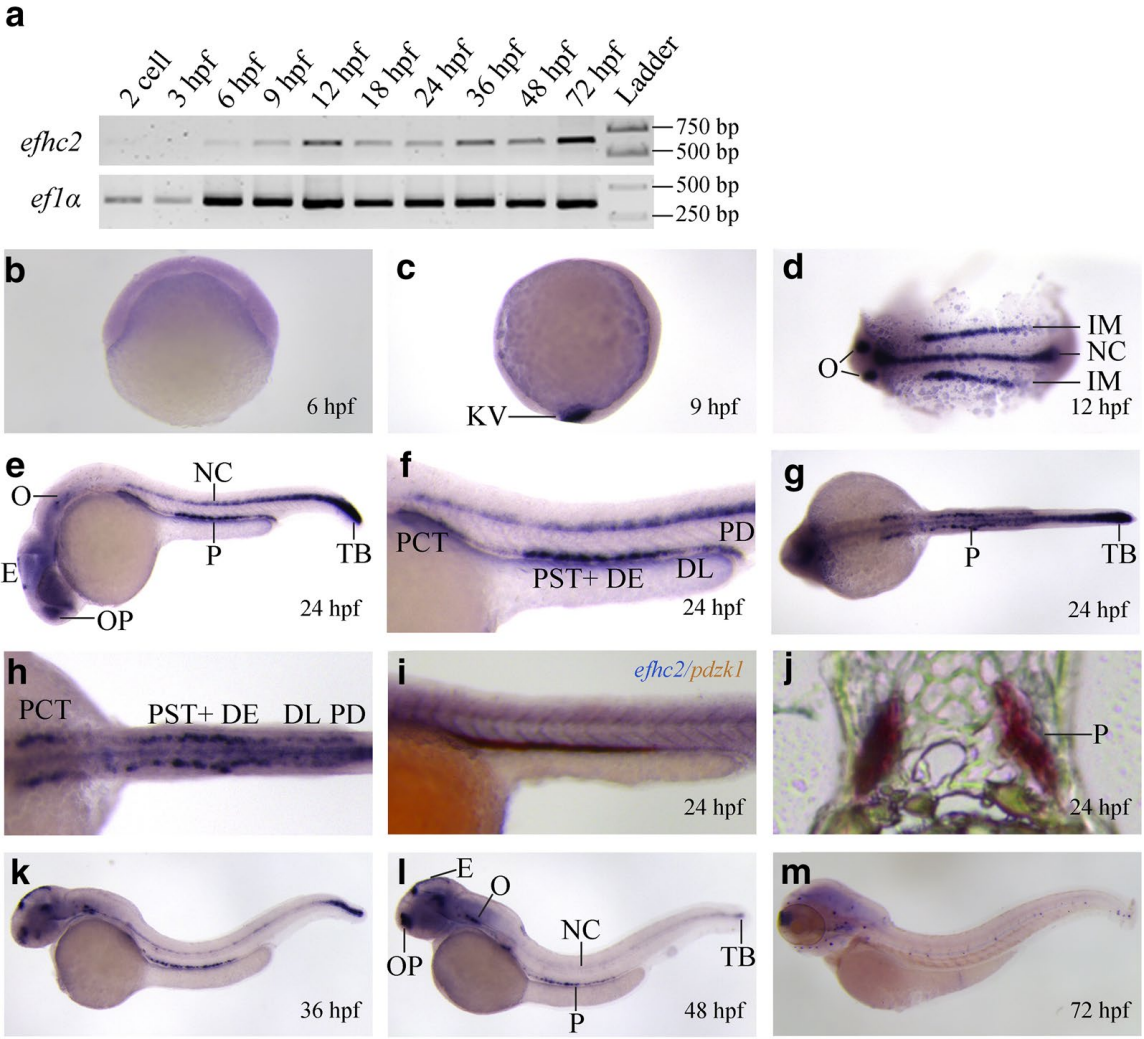
Expression of *efhc2* mRNA during zebrafish development. RT-PCR and whole embryo mRNA in situ hybridization (WISH) was carried out on different stages of zebrafish development. **a** RT-PCR analysis of *efhc2* mRNA expression during zebrafish development. *ef1α* was used as loading control. **b** Ubiquitous expression of *efhc2* at 6 hpf. **c** *efhc2* expression in kupffer’s vesicle (KV) at 9 hpf. **d** Expression in the intermediate mesoderm (IM), notochord (NC) and otic vesicle (O) at 12 hpf. **e** Expression of *efhc2* in olfactory placode (OP), pronephros (P), epiphysis (E), notochord (NC), otic vesicle (O) and in tailbud (TB) at 24 hpf. **f** Magnified image of zebrafish trunk show strong expression of *efhc2* in PST and DE and low expression in PCT, DL and PD. **g** Dorsal view of 24 hpf embryos showing its expression in pronephros. **h**, **i** Two colour WISH of *pdzk1* and *efhc2*. **J** Section through trunk showing *efhc2* expression in pronephros (P). **k**, **l** Expression of *efhc2* in epiphysis, pronephros, notochord, otic vesicle and in tail bud at 36 and 48 hpf. (M) Expression of *efhc2* in neuromast cells and olfactory placode at 72 hpf

Whole-mount mRNA in situ hybridization (WISH) and semi-quantitative RT-PCR were performed to study the spatio-temporal expression pattern of *efhc2* during zebrafish development. Expression of *efhc2* was first observed at 6 hpf by RT-PCR and express during the early development period (Fig. 1a). WISH data showed that *efhc2* mRNA was expressed ubiquitously at 6 hpf (Fig. 1b). By 9 hpf, its expression was localized to kupffer’s vesicle (KV), a transient organ containing ciliated cells (Fig. 1c). At 12 hpf, *efhc2* is expressed in the intermediate mesoderm, notochord and otic vesicle (Fig. 1d). In 24 hpf embryos, *efhc2* is expressed in pronephros, olfactory placode, notochord, otic vesicle, epiphysis, and tail bud. *efhc2* is not expressed in the glomerulus and neck segments of the pronephros, but its expression starts at the PCT. More intense expression of *efhc2* was found in the proximal part of PCT compared to the distal part of the same segment (Fig. 1e, f). Strongest expression of *efhc2* can be seen in the pronephric tubule segments; PST and DE. Its expression is low in DL and PD (Fig. 1e–g). Histological analysis of two colour WISH of *efhc2* and *pdzk1* [7] confirmed the expression of *efhc2* in pronephros (Fig. 1h–j). Both 36 hpf and 48 hpf embryos showed expression of *efhc2* in the pronephros, olfactory placode, notochord, otic vesicle, epiphysis, and tailbud (Fig. 1k, l). At 72 hpf, *efhc2* expression was observed in neuromast cells and olfactory placode (Fig. 1m). Expression pattern indicates the association of *efhc2* with pronephros morphogenesis. Thus, we sought to address the function of *efhc2* in pronephros development in zebrafish.

### Knock-down of Efhc2 reveals its role in pronephros development

**Fig. 2:**
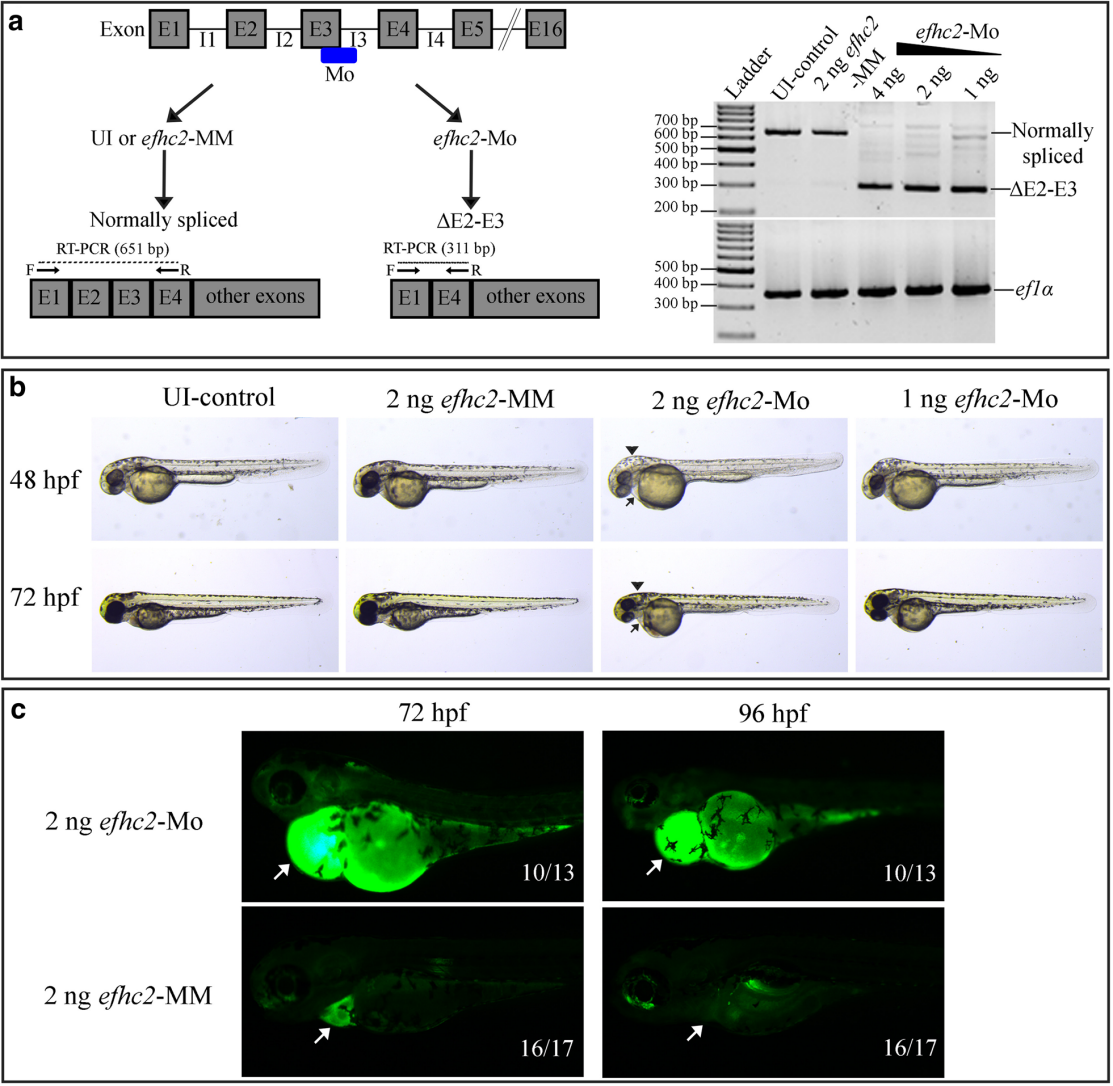
Effect of Efhc2 knock-down on pronephros development and function. **a** Schematic representation of the binding of the splice-blocking *efhc2* morpholino (*efhc2*-MO) targeting exon-3 and the forward and reverse primers used in RT-PCR to verify its effect. RT-PCR on RNA extracted from 24 hpf uninjected embryos or embryos injected with *efhc2*-MM control showed a 651 bp product as expected. The embryos injected with *efhc2*-MO showed a 311 bp fragment confirming the blocking of normal splicing of *efhc2* by this morpholino. **b** *efhc2*-Mo morphants showed morpholino dose-dependent developmental defects as compared with wild-type and mismatch controls. Arrow indicates mild pericardial oedema and arrowhead indicates hydrocephalus. **c** Fluorescent 40 kDa dextran was injected into cardinal vain of control and *efhc2*-Mo morphants at 48 hpf. Accumulation of dextran in yolk and oedema shows defects in pronephros function in *efhc2*-Mo morphants at 72 and 96 hpf

Zebrafish *efhc2* gene contains 16 exons. A morpholino antisense oligo targeting exon-3/intron-3 splice donor junction was designed to block the pre-mRNA splicing of *efhc2* (*efhc2*-Mo). Corresponding mis-match morpholino antisense oligo (*efhc2*-MM) containing 5 mis-matches compared to *efhc2*-Mo that was predicted not to affect normal splicing of *efhc2* pre-mRNA was used as a control. *efhc2*-Mo was injected in different quantities (1–4 ng) into one-cell stage embryos and its effect on RNA splicing was checked at 24 hpf by RT-PCR using forward and reverse primers corresponding to exon-1 and exon-4 respectively. As expected, RT-PCR amplification of *efhc2* cDNA prepared from isolated RNA from 24 hpf wild-type and *efhc2*-MM injected control embryos resulted in a 651 bp product, whereas the *efhc2*-Mo injected embryos showed a 311 bp product (Fig. 2a). Both 651 bp and 311 bp fragments were cloned into pCR-BluntII-Topo vector and sequenced. The sequencing data confirmed that injection of *efhc2*-Mo results in deletion of a 340 bp fragment containing both Exon-2 and 3 (Additional file 1: Figure S1). This indicates that injection of *efhc2*-Mo leads to mis-splicing of *efhc2* mRNA whereas injection of *efhc2*-MM has no effect on normal splicing. Based on above observations, 2 ng of *efhc2*-Mo was injected into embryos for further characterization of its loss-of-function. Same quantity of *efhc2*-MM was used as control. The un-injected or *efhc2*-MM injected embryos did not show any morphological defects (Fig. 2b). The *efhc2*-Mo injected embryos, however, exhibited phenotypic defects such as slightly curved body, mild pericardial oedema and hydrocephalus typically observed in embryos where pronephros development and function is impaired (Fig. 2b). Similar morphological defects were observed when an independent morpholino antisense oligo designed to block *efhc2* translation (*efhc2*-ATG-Mo) was injected (Additional file 2: Figure S2A).

### Knock-down of Efhc2 results in impaired pronephros function

Next, we asked if knock-down of Efhc2 affects the function of the nephrons. Functional zebrafish nephrons of pronephros can easily clear 40 kDa dextran injected into the cardinal vein [16]. The *efhc2*-Mo and *efhc2*-MM morphants were injected with fluorescein isothiocyanate 40 kDa dextran at 48 hpf and the embryos were visualized at 72 and 96 hpf. *efhc2*-MM injected embryos were able to clear it and the dextran was not visible at 72 or 96 hpf. However, abundant fluorescent dextran was seen in embryos where Efhc2 was knocked-down (Fig. 2c). Accumulation of 40 kDa dextran also led to severe pericardial oedema in these embryos (Fig. 2c). These data indicate that Efhc2 is crucial for pronephros function.

### Efhc2 knock-down affects segmentation of distal part of the pronephros

**Fig. 3:**
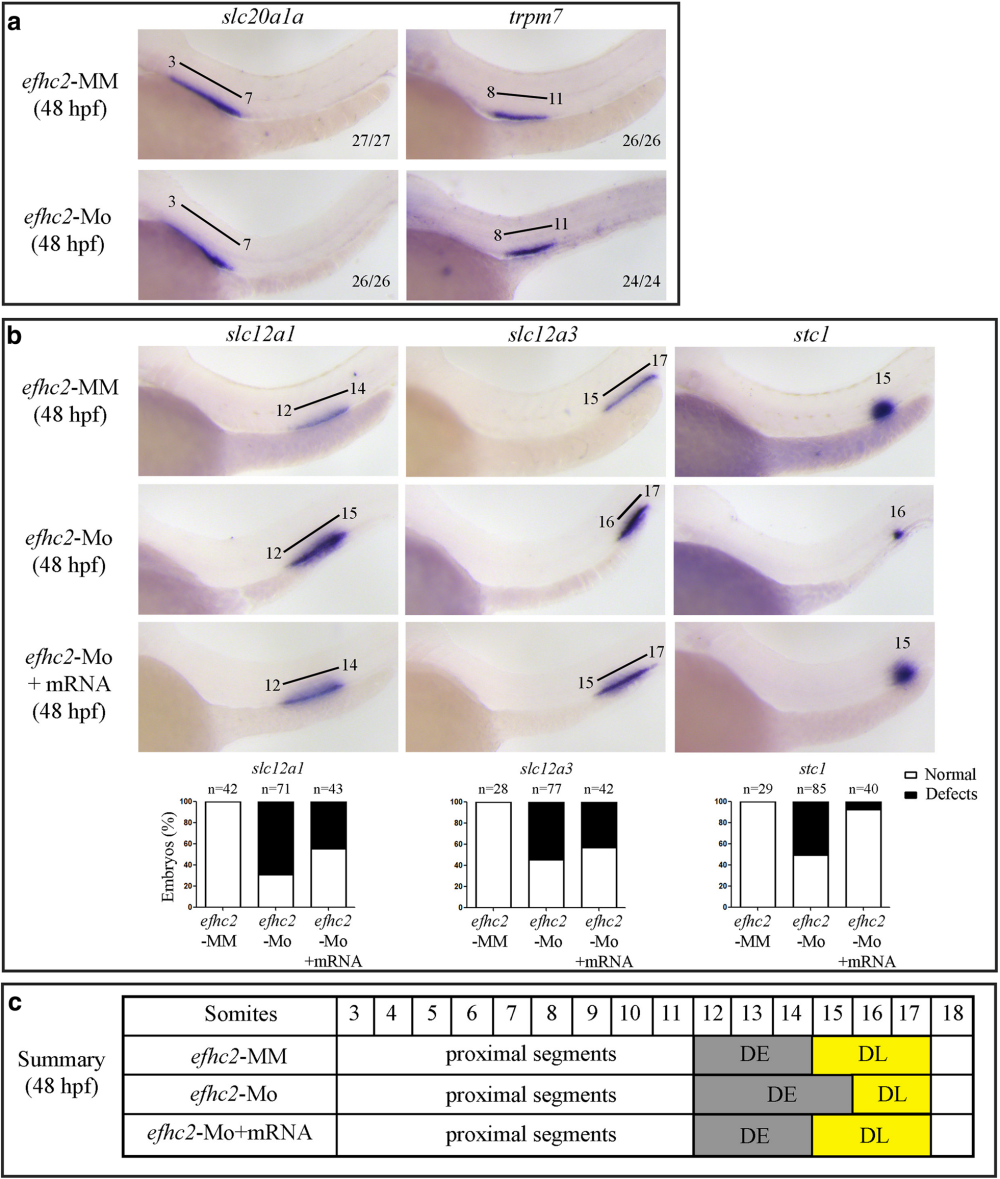
Characterizing the phenotype caused by Efhc2 knock-down. **a** and **b** WISH for pronephros segment specific genes on 48 hpf morpholino injected embryos. Expression of *slc20a1a* (PCT), *trpm7* (PST), *slc12a1* (DE), *slc12a3* (DL) and *stc1* (CS) in *efhc2* mismatch control (*efhc2*-MM) and splice-blocking morpholino (*efhc2*-Mo) injected embryos. The phenotype caused by Efhc2 knock-down was rescued by co-injecting *efhc2* mRNA along with *efhc2* morpholino. **c** Schematic representation of defects caused by Efhc2 knock-down

The nephrons are divided into different segments that reflect their function and are highly conserved among vertebrates [6, 10]. Eight distinct segments can be seen in a nephron of a zebrafish embryo [7]. We asked if loss-of-Efhc2 leads to aberrant segmentation of the nephrons. Sodium-dependent phosphate transporter *slc20a1a* is expressed in the pronephros aligning with 5th to 8th somite at 24 hpf and from 3rd to 7th somite at 48 hpf demarcating the PCT. Transient receptor potential cation channel gene *trpm7* is expressed in part of the nephron adjacent to 9th to 11th somite at 24 hpf and from 8th to 11th somite demarcating the PST segment of the pronephros. The expression domains of *slc20a1a* and *trpm7* were identical in both *efhc2*-Mo and *efhc2*-MM injected embryos (Fig. 3a and Additional file 3: Figure S3A). This indicates that knock-down of Efhc2 does not influence segmentation of the proximal part of the pronephric tubule consisting of PCT and PST. The expression of sodium/potassium/chloride transporter *slc12a1* in the pronephros next to 12th and 13th somite at 24 hpf and 12th to 14th somite in 48 hpf demarcates the DE segment of the nephron. This expression domain of *slc12a1* was not changed in *efhc2*-MM injected embryos. However, Efhc2 knock-down led to the expression of *slc12a1* in 12th to 15th somite in both 24 and 48 hpf embryos (Fig. 3b and Additional file 3: Figure S3A). This expression domain of *slc12a1* in Efhc2 knock-down embryos indicates that loss-of-Efhc2 function results in the expansion of DE distally. The DE expansion was observed in 53% (51/97) at 24 hpf and 69% (49/71) at 48 hpf in Efhc2 knock-down embryos (Fig. 3b and Additional file 3: Figure S3A). A second morpholino designed to block *efhc2* translation (*efhc2*-ATG-Mo) also lead to expansion in DE expression domain by 3 somites at 48 hpf in 59% (16/27) embryos as marked by *slc12a1* expression (Additional file 2: Figure S2B).

The *efhc2*-Mo morphants showed significantly reduced DL (Fig. 3b and Additional file 3: Figure S3A). The expression of DL marker sodium/chloride transporter *slc12a3* was confined to pronephros adjacent to 16th and 17th somite in 24 hpf and 48 hpf embryos injected with *efhc2*-Mo. Whereas, the position of DL segment was normal in *efhc2*-MM injected embryos, where it was expressed next to 14th to 17th somite in 24 hpf and 15th to 17th somite in 48 hpf embryos (Fig. 3b and Additional file 3: Figure S3A). The DL reduction in *efhc2*-Mo morphants was 53% (54/102) at 24 hpf and 55% (42/77) at 48 hpf (Fig. 3b and Additional file 3: Figure S3A). This result was confirmed by injection of *efhc2*-ATG-Mo, which resulted in reduction of DE domain in morpholino injected embryos. The *slc12a3* expression domain was reduced to 16th and 17th somite at 48 hpf in 59% (16/27) embryos as compared to normal expression from 15th to 17th somite (Additional file 2: Figure S2B). Stanniocalcin (*stc1*) is expressed in CS (next to somite number 15) and is involved in calcium and phosphate homeostasis. The expression level and position of *stc1* was affected by knock-down of Efhc2. *stc1* expression was reduced in *efhc2*-Mo injected embryos as compared to embryos injected with control *efhc2*-MM morpholino. Its localization was shifted distally next to 16th somite, whereas *efhc2*-MM injected embryos showed normal expression of *stc1* adjacent to 15th somite. The CS reduction in *efhc2*-Mo morphants was 50% (43/85) (Fig. 3b and Additional file 3: Figure S3A). *efhc2*-ATG-Mo injected embryos also showed the same results as seen with *efhc2*-Mo injected embryos. The expression level of *stc1* was reduced and the expression domain was sifted from 15th somite to 16th somite in *efhc2*-ATG-Mo injected embryos (Additional file 2: Figure S2B). To confirm the specificity of phenotype caused by morpholino-mediated knock-down of Efhc2, we co-injected zebrafish *efhc2* mRNA with *efhc2*-Mo. To check overexpression phenotype, we injected 100–300 pg of *efhc2* mRNA/embryo. Embryos injected with 100–200 pg mRNA develop normally. However, more than 200 pg mRNA injection leads to severe developmental defects (Additional file 4: Figure S4). Hence, we have injected 200 pg *efhc2* mRNA for rescue experiments. Co-injection of zebrafish *efhc2* mRNA partially rescued the effect of *efhc2*-Mo mediated knock-down of endogenous Efhc2. We found that the DE segment defect was rescued by 62.5% (20/32) at 24 hpf and 56% (24/43) at 48 hpf. DL segment defect was rescued by 62.07 (18/29) at 24 hpf and 57% (24/42) embryos at 48 hpf. More than 92.5% of *efhc2*-Mo morphants were rescued for CS segment development (Fig. 3b, c). Thus, our data suggests that the pronephros segmentation phenotype is Efhc2 knock-down specific.

Taken together, our experiments suggest that loss-of-Efhc2 function leads to expansion of DE distally and reduction of CS and DL segments of the distal pronephric tubule in zebrafish embryos (Fig. 3, Additional file 2: Figure S2 and Additional file 3: Figure S3). Hence, Efhc2 is essential for normal segmentation of the distal part of the pronephros.

### Efhc2 has no influence on Retinoic acid mediated segmentation of zebrafish pronephros

**Fig. 4:**
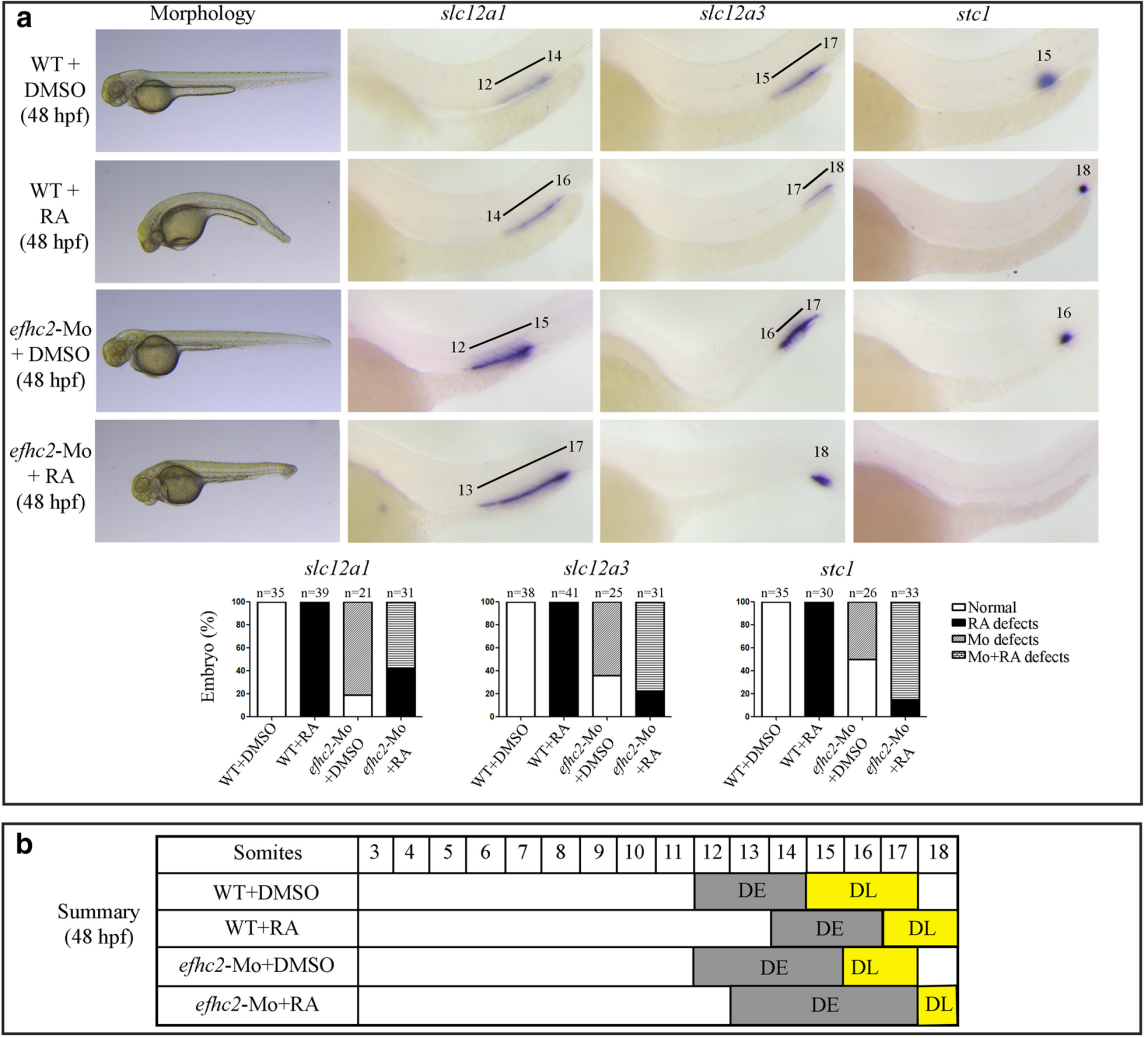
Role of Efhc2 in RA mediated segmentation of pronephros development. **a** Wild-type embryos or *efhc2* morphants were treated with RA. Morphological changes caused by RA treatment in wild-type and *efhc2* morphants. WISH showing expression of *slc12a1* (DE), *slc12a3* (DL) and *stc1* (CS). Wild-type embryos or *efhc2* morphants were treated with RA (1 × 10^−7^ M). *efhc2* morphants treated with RA show expansion of expression domain of DE marker *slc12a1* and almost or complete loss of DL and CS markers *slc12a3* and *stc1* as compared with wild-type embryos treated with RA. **b** Summary of the effects of RA on WT and *efhc2* morphants at 48 hpf

Our findings reveal an important role for Efhc2 in the segmentation of distal part of zebrafish pronephros. Several reports suggest that RA signaling is important for nephron segmentation in vertebrates [4, 7]. RA signaling is required for the development of PCT and PST and is thought to inhibit the formation of distal segments such as DE and DL in zebrafish [15]. Exogenous treatment of RA results in a pronephros with expanded PCT and PST. The DE and DL segments are either reduced or shifted distally dependent on the time and concentration of RA treatment [7]. Conversely, inhibition of RA synthesis by DEAB (4-diethylaminobenzaldehyde) results in reduced proximal segments and expanded distal segments [7]. Hence, we asked if Efhc2 plays any role in RA mediated inhibition of distal pronephric segment formation. The uninjected control or *efhc2*-Mo injected embryos were treated with exogenous RA (1 × 10^−7^ M) from 9 to 16 hpf and the formation of distal pronephric tubule segments were checked at 24 and 48 hpf. As reported by other groups, RA treated wild-type embryos had the DE segment shifted distally compared to vehicle (DMSO) treated embryos. The RA treated embryos expressed DE marker *slc12a1* in a domain next to 14th to 16th somite compared the DMSO treated controls that showed normal expression domain next to 12th to 14th somite. Injection of *efhc2*-Mo did not shift the DE segment and the anterior domain of *slc12a1* was still localized to 12th somite. These embryos, however, had a slightly expanded DE segment. The DE segment shifted distally and expanded when *efhc2*-Mo injected embryos were treated with RA (Fig. 4a and Additional file 5: Figure S5). Essentially, RA treatment of Efhc2 knock-down embryos exhibited a phenotype that was a combination of both RA treatment and Efhc2 knock-down (Fig. 4a, b).

Next, we examined the effect of RA treatment on DL and CS segments in embryos lacking Efhc2. DMSO treated wild-type embryos showed expression of DL marker *slc12a3* next to 15th to 17th somite, which was shifted distally and reduced to an expression domain next to 17th and 18th somite when treated with RA. The *efhc2*-Mo injected embryos had the *slc12a3* expression domain reduced in nephron next to 16th and 17th somite. However, when these Efhc2 knock-down embryos were subjected to RA treatment, the DL segment was dramatically reduced to only one somite length and occupied the distal most part of the pronephric tubule next to 18th somite (Fig. 4a and Additional file 5: Figure S5). RA treatment of embryos lacking Efhc2 had a similar effect on the CS. The CS segment marker *stc1*, which is normally expressed next to 15th somite, was reduced and shifted distally to 18th somite in the RA treated and 16th somite in *efhc2*-Mo injected embryos. This expression of *stc1* was completely abolished in more than 90% embryos that lacked Efhc2 and were treated with RA (Fig. 4a).

In summary, *efhc2*-Mo injected embryos show two distinct and opposing effects in response to exogenous RA treatment within the distal pronephric tubule segments. RA treatment of embryos lacking Efhc2 function expands the DE segment whereas, under same conditions, the CS and DL segments are much reduced (Fig. 4b and Additional file 5: Figure S5). However, the effect of RA treatment of Efhc2 knock-down embryos reflects a combination of the effects of Efhc2 knock-down and exogenous RA treatment. Hence, the effect of RA and Efhc2 on pronephros segmentation may be independent of each other.

**Fig. 5:**
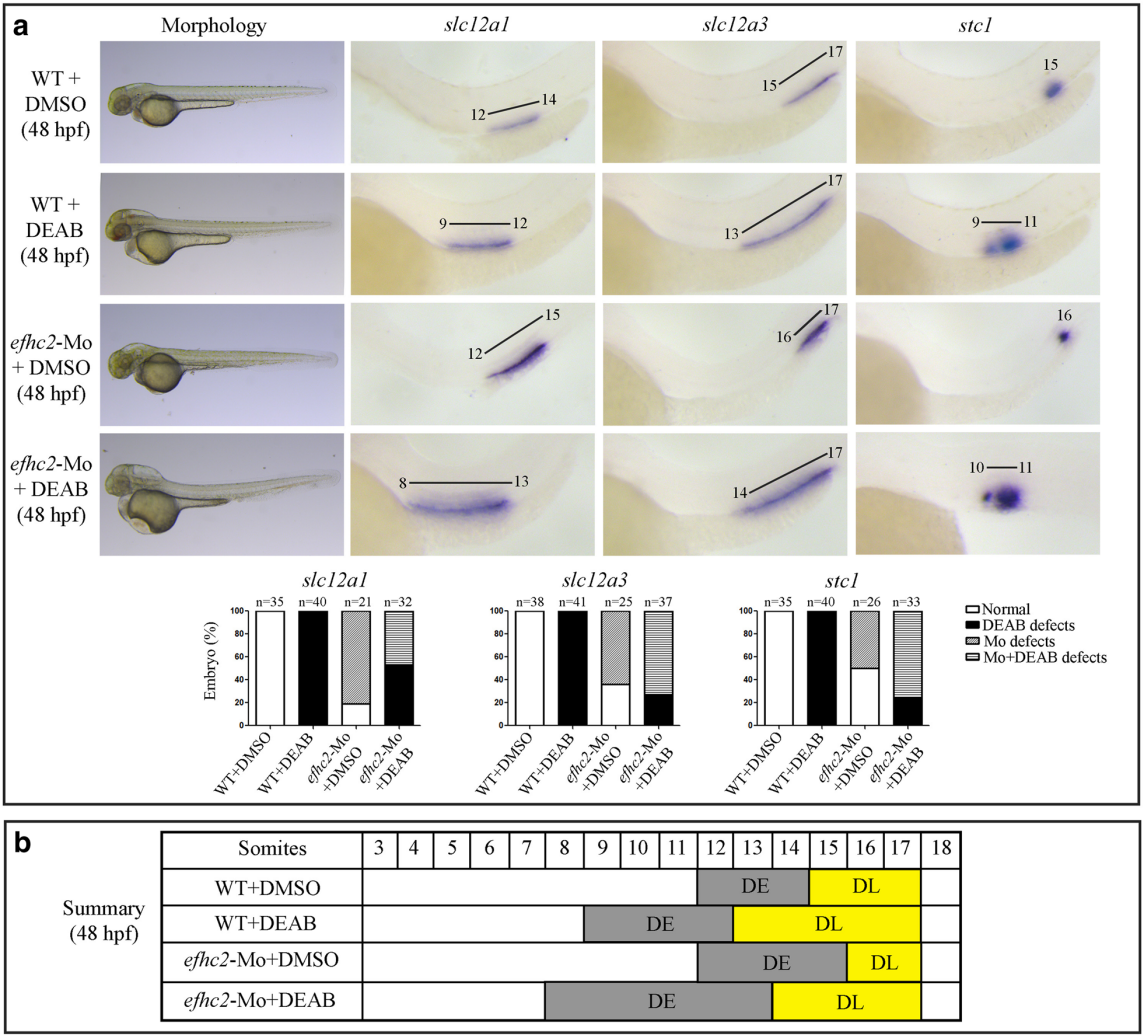
Effect of blocking RA synthesis by DEAB in *efhc2*-Mo morphants during pronephros segmentation. **a** Wild-type embryos or *efhc2*-Mo morphants were treated with DEAB (1 × 10^−5^ M). Morphological changes caused by DEAB treatment in wild-type and *efhc2* morphants. *efhc2*-Mo morphants treated with DEAB show expansion of expression domain of DE marker *slc12a1* and slight reduction of DL and CS markers *slc12a3* and *stc1* as compared with wild-type embryos treated with DEAB. **b** Summary of the effects of DEAB on WT and *efhc2*-Mo morphants at 48 hpf

**Fig. 6:**
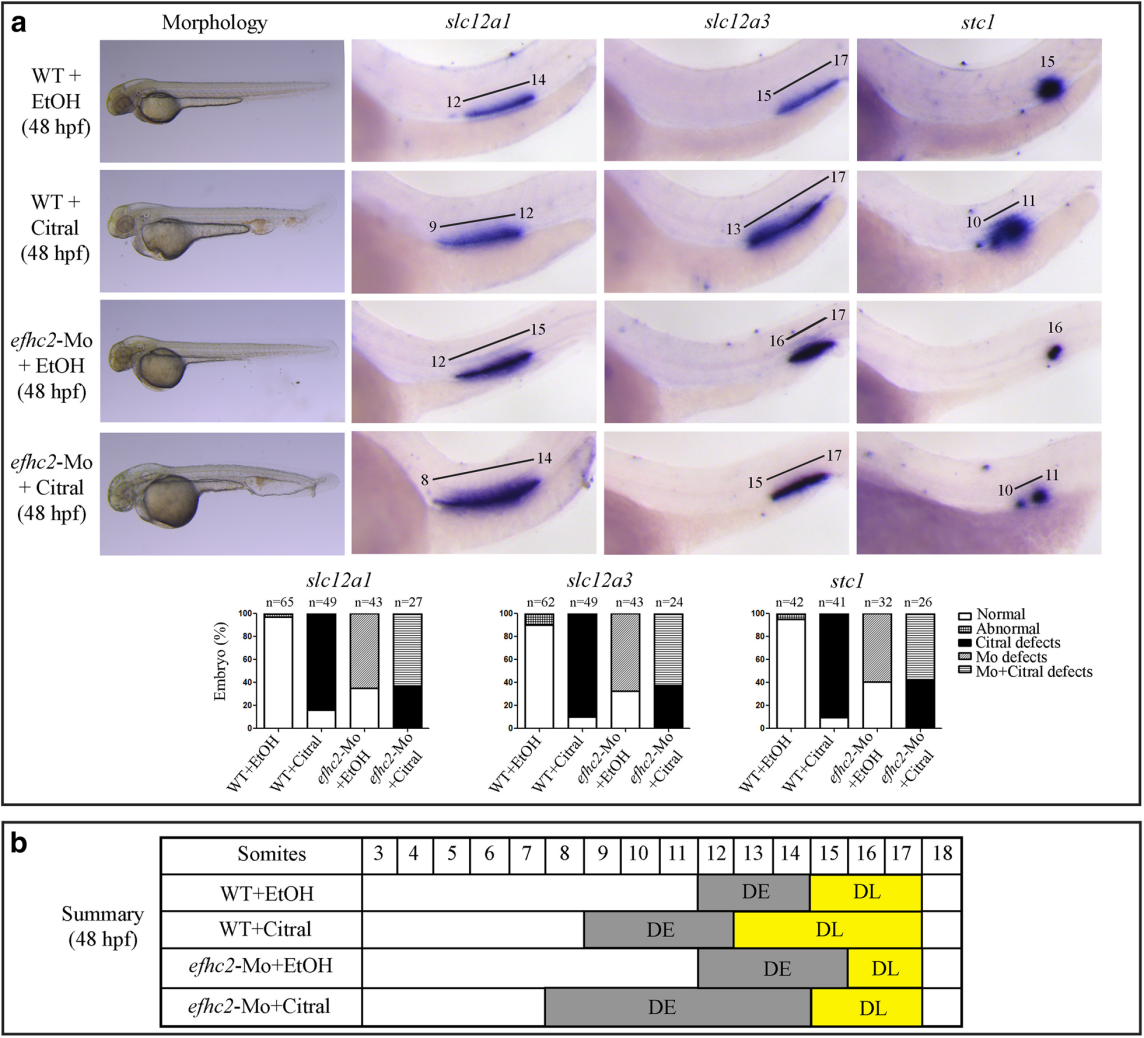
Effect of RA synthesis blocker citral on pronephros segmentation in *efhc2*-knocked-down embryos. **a** Wild-type embryos or *efhc2*-Mo morphants were treated with citral (2.5 × 10^−5^ M). Morphological defects caused by citral treatment in wild-type and *efhc2* morphants. *efhc2*-Mo morphants treated with citral show expansion of expression domain of DE marker *slc12a1* and slight reduction of DL and CS markers *slc12a3* and *stc1* as compared with wild-type embryos treated with citral. **b** Summary of the effects of citral on WT and *efhc2*-Mo morphants at 48 hpf

Next, we checked the effect of lack of RA signaling on Efhc2 mediated segmentation of distal part of the nephron. RA synthesis was inhibited by treatment of embryos with 4-diethylaminobenzaldehyde (DEAB) (1 × 10^−5^ M) or 3,7-dimethyl-2,6-octadienal (citral) [17] (2.5 × 10^−5^ M) from 9 to 16 hpf, and the effect of this treatment on pronephros segmentation was monitored at 24 and 48 hpf using segment-specific marker gene expression. DEAB treatment resulted in nephrons that had the DE domain shifted proximally and the DE marker *slc12a1* was expressed in a region next to 9th to 12th somite as compared to its expression next to 12th to 14th somite in vehicle (DMSO) treated embryos (Fig. 5a). However, DEAB treatment of Efhc2 knock-down embryos resulted in *slc12a1* expression in nephron next to 8th to 13th somite (Fig. 5a and Additional file 5: Figure S5). Blocking RA synthesis by citral also resulted in similar changes in DE marker expression. Citral treatment of Efhc2 knock-down embryos resulted in *slc12a1* expression in nephron next to 8th to 14th somite (Fig. 6a). This indicates that inhibition of RA synthesis by DEAB or citral in combination with Efhc2 knock-down leads to embryos with expanded DE segment as seen in *efhc2*-Mo morphants and a slight shift of this domain to proximal part of the embryos as seen in DEAB or citral treated embryos (Figs. 5b and 6b).

Inhibition of RA signaling by DEAB or citral results in expansion and slight proximal shifting, and Efhc2 knock-down results in reduced DL segment. The DL marker *slc12a3* was expressed next to 13th to 17th somite in DEAB or citral treated embryos as compared to its normal expression domain of 15th to 17th somite in vehicle treated embryos. *slc12a3* was expressed in 16th to 17th somite in Efhc2 knock-down embryos (Figs. 5a, 6a and Additional file 5: Figure S5). DEAB treatment of Efhc2 knock-down embryos resulted in *slc12a3* expression in 14th to 17th somite. Citral treatment of Efhc2 knock-down embryos resulted in *slc12a3* expression in nephron next to 15th to 17th somite. This indicates that DEAB or citral treatment of Efhc2 knock-down embryos had resulted in DL segment that had shifted proximally compared to both wild type or Efhc2 knock-down embryos. The DL segment in these embryos was expanded when compared to vehicle treated or Efhc2 knock-down, but there was a reduction in DL domain when compared to DEAB or citral treatment alone. These effects of RA signaling inhibition on DE and DL in Efhc2 knock-down embryos is opposite of the effects seen upon exogenous RA treatment. These data support the observation that exogenous RA treatment or inhibition of RA synthesis is able to exhibit their effects in absence of Efhc2.

The CS segment present next to 15th somite was shifted proximally and expanded upon DEAB treatment. Expression of CS marker *stc1* was much reduced and was shifted distally to 16th somite after Efhc2 knock-down. DEAB treatment of embryos lacking Efhc2 resulted in enhanced *stc1* expression in somite 10th to 11th (Fig. 5a and Additional file 5: Figure S5). Citral treatment of Efhc2 knock-down embryos also resulted in enhanced expression of *stc1* in nephron next to 10th to 11th somite (Fig. 6). This effect of DEAB or citral on *efhc2*-Mo morphants is exact opposite of RA treatment of same embryos where *stc1* expression was completely abolished. Taken together, these results indicate that RA signaling is able to control the formation of CS in absence of Efhc2. Hence, although both RA and Efhc2 affect distal segmentation of pronephros, their functions are not interdependent.

### Efhc2 is required for formation of multi-ciliated cells (MCC)

**Fig. 7:**
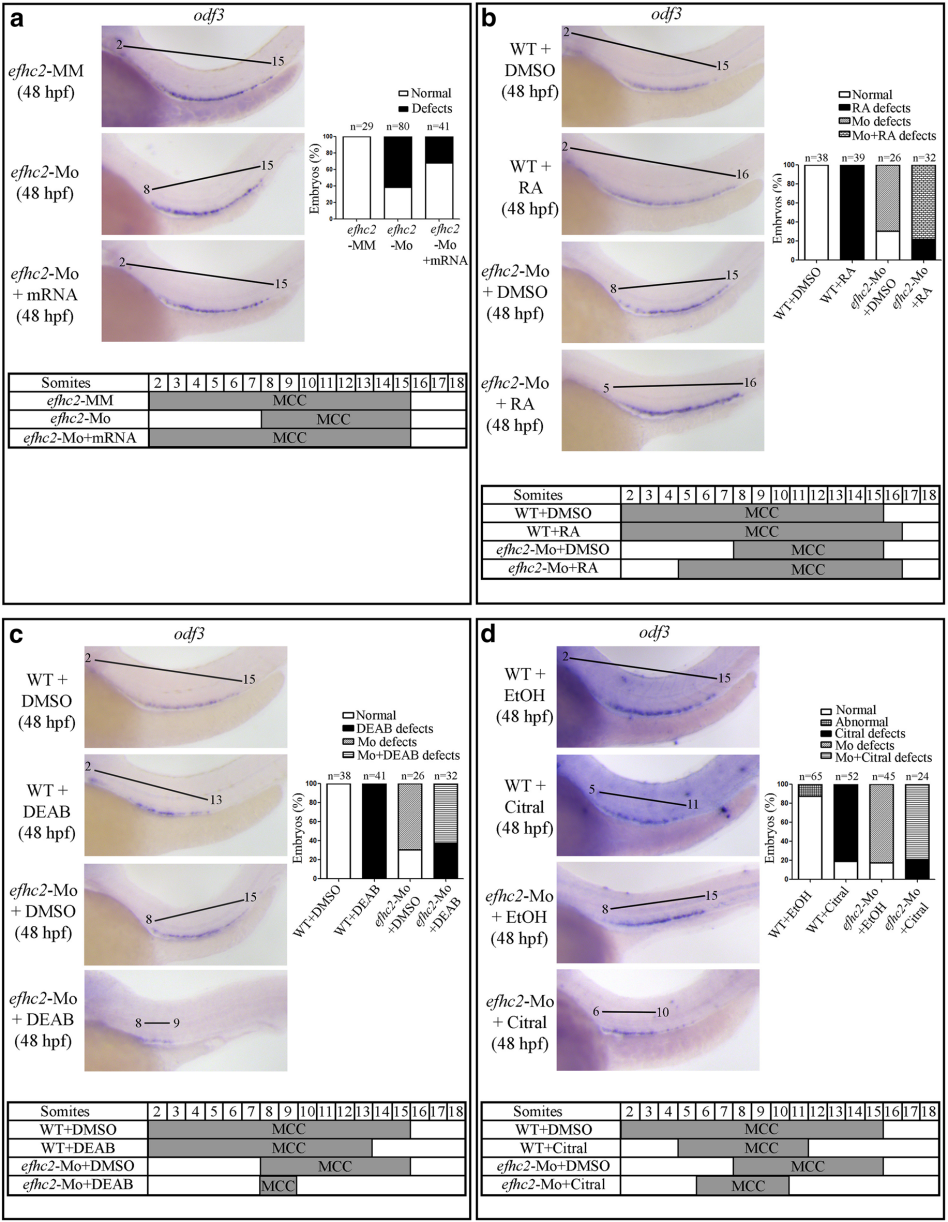
Role of Efhc2 and RA in MCC development. **a** WISH for MCC specific marker *odf3* in *efhc2* mismatch control (*efhc2*-MM) and splice-blocking morpholino (*efhc2*-Mo) injected embryos. The phenotype caused by Efhc2 knock-down was rescued by *efhc2* mRNA. **b** Wild-type embryos or *efhc2*-Mo morphants were treated with RA. WISH showing expression of *odf3* gene. *efhc2*-Mo morphants treated with RA show reduction of expression domain of MCC as compared to wild-type embryos treated with RA. **c** Wild-type embryos or *efhc2*-Mo morphants were treated with DEAB. WISH showing expression of *odf3* gene. *efhc2*-Mo morphants treated with DEAB show reduction of expression domain of MCC as compared to wild-type embryos treated with DEAB. **d** Wild-type embryos or *efhc2*-Mo morphants were treated with citral. WISH showing expression of *odf3* gene. *efhc2*-Mo morphants treated with citral show reduction of expression domain of MCC as compared to wild-type embryos treated with citral

Multi-ciliated cells (MCC) are present along the PCT, PST, DE and anterior part of DL segments of pronephros in zebrafish embryos [16]. These cells can be identified by WISH using ciliogenesis genes such as *odf3* and *rfx2* [18]. We asked if knock-down of Efhc2 had any effect on MCC formation. The control *efhc2*-MM injected embryos expressed *odf3* corresponding to 2nd to 15th somite, whereas the *efhc2*-Mo morphants had *odf3* expression next to 8th to 15th somite (Fig. 7a). This suggests that embryos injected with *efhc2*-Mo had a much-reduced domain of *odf3* expression compared to embryos injected with mis-match control. The MCC reduction in *efhc2*-Mo morphants was 59% (63/106) at 24 hpf and 61% (49/80) at 48 hpf (Fig. 7a and Additional file 3: Figure S3). This effect of Efhc2 knock-down was rescued by co-injection of *efhc2* mRNA in 68.3% (26/33) embryos at 48 hpf (Fig. 7a). Injection of *efhc2*-ATG-Mo also lead to reduction in MCC formation. Fifty five percent embryos (21/38) had the *odf3* expression level and domain reduced (Additional file 2: Figure S2). These observations indicate that Efhc2 positively regulates pronephric multi-ciliated cell development (Fig. 7a and Additional file 2: Figure S2).

**Fig. 8:**
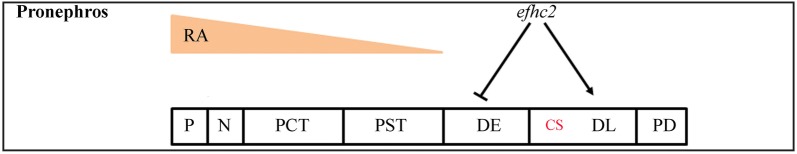
Model of Efhc2 function during zebrafish pronephros development. RA signaling induces proximal and inhibits expansion of distal segments. Efhc2 is important for the segmentation of distal part of the pronephros. Efhc2 may act independently of RA signaling in the segmentation of pronephros

It is known that RA signaling promotes MCC formation [16]. Exogenous treatment of RA enhances MCC number and these cells are expressed slightly distally in response to RA treatment. Conversely, treatment of embryos with RA inhibitor DEAB reduced MCC number. Knock-down of Efhc2 resulted in reduced MCC number and the expression domain of MCC marker *odf3* was also reduced. Hence, we asked if Efhc2 plays any role in RA mediated MCC formation. The wild-type and Efhc2 knock-down embryos were treated with RA (1 × 10^−7^ M) or RA inhibitors DEAB (1 × 10^−5^ M) or citral (2.5 × 10^−5^ M) from 9 to 16 hpf and development of MCC in these embryos were examined using the expression of MCC marker *odf3* at 48 hpf. The RA treated wild-type embryos showed an increase in MCC number and expansion *odf3* expression domain as compared to DMSO treated embryos (Fig. 7b). The expression of *odf3* is seen in pronephros adjacent to 2nd to 16th somite in 48 hpf RA treated embryos. However, *efhc2*-Mo injected embryos treated with same concentration of RA showed expression of *odf3* next to 5th to 16th somite in 48 hpf embryos as compared to expression of *odf3* from 8th to 15th somite in Efhc2 knock-down alone (Fig. 7b and Additional file 5: Figure S5). This indicates that RA was able to partially compensate for loss-of-Efhc2 function in the development of MCC. As compared with RA treated embryos, wild-type embryos treated with DEAB showed reduced MCC formation (Fig. 7c and Additional file 5: Figure S5). The expression of *odf3* is seen adjacent to 2nd to 13th somite in 48 hpf embryos as compared to DMSO treated embryo, where its expression can be seen from 2nd to 15th somite (Fig. 7c). *efhc2*-Mo morphants treated with DEAB show almost or complete loss of *odf3* expressing multi-ciliated cells (Fig. 7c and Additional file 5: Figure S5). Treatment of Efhc2-knock-down embryos with another RA inhibitor citral, also lead to similar reduction of number and domain of *odf3* expressing MCC (Fig. 7d). This indicates that the inhibition of RA synthesis by DEAB or citral leads to a dramatic loss of MCC formation in *efhc2*-Mo injected embryos. This could be interpreted as loss of Efhc2 and RA signaling has a synergistic effect on reduced MCC formation. Taken together, our results suggest that RA and Efhc2 synergistically regulate MCC development (Fig. 8).

## Discussion

Here, we have identified Efhc2 as a regulator of nephron segmentation in zebrafish. Knock-down of Efhc2 specifically affects the segmentation of distal pronephric tubule consisting of DE, CS, and DL. Development of the proximal tubule segments such as PCT and PST was largely unaffected in these embryos. The expression of *efhc2* starts in a non-spatially restricted manner by 6 hpf. First localized expression of *efhc2* can be seen at 9 hpf in kupffer’s vesicle (KV). Kupffer’s vesicle (KV) is a transiently present organ that contains cilia and has been shown to establish asymmetric cell signaling and gene expression [19]. In zebrafish, kupffer’s vesicle (KV), pronephros, otic vesicle, olfactory placode and neuromast cells are the organs which have abundant cilia [20]. *efhc2* expression in these organs indicates that Efhc2 may have a role in cilia development or function. Consistent with this hypothesis, we observe that Efhc2 is required for formation of the multi-ciliated cells (MCC) in the pronephros. It has been shown that DM10 domain-containing proteins in *Chlamydomonas* are bound to flagellar microtubules and are proposed to be involved in axonemal targeting and assembly [21]. Efhc2, which contains three DM10 domains [22], may have a similar role in assembly and stability of cilial axoneme [23].

The expression of *efhc2* mRNA was not uniform in the PCT segment. The proximal part of the PCT had more intense expression of *efhc2*, whereas, the distal part had very low expression. Unlike zebrafish proximal tubule segments, which is divided into PCT and PST, the mammalian proximal tubule is divided into 3 segments (S1, S2 and S3) [10]. The *Xenopus* proximal segment is also thought to consist of 3 different segments (PT1, PT2 and PT3) [24]. It is not clear if the distal part of PCT lacking *efhc2* expression represents a segment similar to PT2 and S2 seen in *Xenopus* and mammals respectively. *efhc2* expression could not be detected in pronephros in 72 hpf embryos, which could be due to low penetrance of the anti-sense probe at this stage. However, the expression of this gene in the neuromast cells was clearly visible. These mechanosensory cells are responsible for sensing water movement and contain cilia [25]. Knock-down of Efhc2 showed only mild morphological defects in zebrafish embryos. However, there was a significant impact of its loss-of-function on the segmentation of pronephros. Paired box 2 and 8 (Pax2a and Pax8) act early during development in the intermediate mesoderm to specify pronephros. Hnf1ba/b act downstream of Pax2a/8 to divide the pronephric region to rostral, central and caudal regions that give rise PCT, PST, DE and DL segments [13]. Hnf1ba/b transcription factors activate Irx3b in 10–15 somite zebrafish embryos that specify the PST and DE segments. However, in 15–28 somite embryos, Irx3b acts upstream of Hnf1ba/b to maintain its expression and specification of DE segment, whereas Hnf1ba alone is sufficient for maintaining DL segment [13]. RA signaling plays a major role is pronephros segmentation [6, 7]. Our data suggests that Efhc2 acts independent of RA signaling in segmentation of distal nephric regions DE and DL. It would be interesting to see if Efhc2 acts downstream of transcription factors such as Hnf1ba/b. However, the effect of Efhc2 and RA signaling on MCC formation was synergistic. Hence, Efhc2 may have RA dependent and independent roles during zebrafish development.

Zebrafish *efhc1* and *efhc2* are paralogous genes with difference in the presence or absence of a C-terminal Ca^2+^ binding EF-hand domain. In zebrafish, Efhc1 does not have a Ca^2+^ binding EF-hand domain but Efhc2 has this domain. Deletion studies of *Xenopus* Efhc1b show that, all three DM10 domains are expressed in the ciliary axonemes. The expression of Wnt8a is increased in absence of Efhc1b in *Xenpous*, indicating its role in regulating Wnt signaling pathway which is crucial for normal development [23]. In zebrafish, both *efhc2* (in this study) and *efhc1*, are expressed in the pronephros. We show that zebrafish Efhc2 is required for segmentation of the distal part of pronephros. It will be of interest to identify the role of Efhc1 during zebrafish, particularly in pronephros development and function.

In summary, we have identified a novel component of genetic programme regulating pronephros segmentation in zebrafish. We find that Efhc2 regulates the distal segments of pronephros, the DE, CS, and DL. Efhc2 is expressed in ciliated cells and our work reveals a role for this gene in MCC development.

## Conclusions

Our study has uncovered the role of *efhc2* gene in the segmentation of distal part of the pronephros. We have also shown that RA and Efhc2 work independently in the process of pronephros segmentation, whereas they work synergistically in MCC development.

## Methods

### Zebrafish husbandry

Zebrafish wild-type strains Albino and TÜbingen (TÜ**)** were used in all experiments. The experiments carried out were approved by the institutional animal ethics committee. The embryos were staged according to Kimmel et al. [26].

### Zebrafish whole embryo in situ hybridization (WISH)

Embryos at different stages [26] were fixed in 4% PFA and stored in 100% methanol at − 20 °C. Whole-mount in situ hybridization was performed as previously described [27, 28]. Riboprobes against zebrafish *efhc2*, *slc20a1a*, *trpm7*, *slc12a1*, *slc12a3*, *stc1*, *odf3* and *pdzk1* were synthesized and used. The DIG or Fluorescein labelled RNA probes were synthesized by linearizing the plasmids and transcribing with T7/SP6 RNA polymerases (*efhc2*, *stc1*and *pdzk1* plasmids were linearized with *Xho*I and transcribed with SP6, *slc20a1a* linearized with *Xba*I and transcribed with SP6, *trpm7* linearized with *Sac*II and transcribed with SP6, *slc12a1* linearized with *Kpn*I and transcribed with T7, *slc12a3* and *odf3* plasmids were linearized with *Bam*HI and transcribed with T7. BM-purple and INT-BCIP (Roche) were used as chromogenic substrates to visualize the expression. Pictures were taken using Leica MZ16 stereo microscope.

### Morpholino and mRNA injection

The antisense morpholino oligonucleotide was obtained from Gene Tools. The *efhc2* splice-blocking morpholino (*efhc2*-Mo, 5′ GTTTGATTCTGATGGTTCACCTTGT-3′) was designed targeting exon3/intron 3 splice donor junction and a mismatch morpholino (*efhc2*-MM) 5′ GTaTcATTCTcATGcTTCACCTTcT-3′ with five base mismatches (small letters) was used as a control. The *efhc2*-ATG-Mo (5′ TTCCAGGCAGCATTGGTAACGCCAT-3′) was designed to block translation of *efhc2* mRNA. Morpholino was dissolved in nuclease-free water as 1 mM stock. 1 or 2 ng of morpholino in 1 nl 1X Danieau solution [58 mM NaCl, 0.7 mM KCl, 0.4 mM MgSO_4_, 0.6 mM Ca(NO_3_)_2_, 5 mM HEPES pH 7.6] was injected into 1–2 cell stage embryos using a Femtojet microinjector (Eppendorf).

For rescue experiments, full-length zebrafish *efhc2* (2257 bp) was amplified by PCR. The forward primer contains *Eco*RI and the reverse primer contains *Xho*I sites. The *efhc2*-PCR amplicon was cloned into a pCR-BluntII-Topo vector (Invitrogen) and was then sub-cloned into a pCS2 + vector at the *Eco*RI and *Xho*I sites. To synthesize *efhc2* mRNA, the *efhc2*-pCS2 vector was linearized with *Not*I and transcribed using SP6 mMessage mMachine kit (Ambion). To determine the optimal dose for rescue, 100, 200 or 300 pg *efhc2* mRNA was co-injected with 2 ng *efhc2*-Mo morpholino. The optimal dose for rescue was found to be 200 pg.

### RT-PCR

To check the efficiency of *efhc2* splice-blocking morpholino, total RNA was isolated from embryos injected with *efhc2*-Mo and control embryos injected with *efhc2*-MM. An equal amount of RNA was used to synthesize cDNA using superscriptIII kit (Invitrogen). The primers (flanking *efhc2* exon 1 and exon 4) and PCR conditions were as follows: (forward 5′-CTGTCGTCCAACTGAGGGAAA-3′, reverse 5′-CTCTTGTCGAAGGGTAGTATAGGG-3′, Tm 55 °C, 30 cycles). Same PCR primers were used for amplifying *efhc2* from different developmental stages of zebrafish.

### Chemical treatment

Retinoic acid (10 mM) and DEAB (1 M) (Sigma-Aldrich) were dissolved in 100% dimethyl sulfoxide (DMSO), aliquoted and stored at − 80 °C. Citral was dissolved in 100% ethanol. Briefly, embryos were incubated in dark from 9 to 16 hpf stage in 1 × 10^−7^ M RA/DMSO, 1 × 10^−5^ M DEAB/DMSO and 2.5 × 10^−5^ M citral/ethanol made with E3 embryo media. For control, embryos were incubated with either DMSO or ethanol. Embryos were fixed at 24 and 48 hpf and analysed by WISH.

### Dextran injection

To check the effect of Efhc2 knock-down on zebrafish pronephros function, we injected 40 kDa fluorescein isothiocyanate dextran (Sigma) in common cardinal vein of 48 hpf embryos [16]. Clearance of dextran was analysed at 72 hpf and 96 hpf.

### Histology

WISH stained embryos were fixed in 4% PFA for 1–2 h at room temperature, washed with PBS and then transferred into 30% sucrose in PBS for overnight. Next day, embryos were incubated in a 1:1 mixture of OCT and 30% sucrose/PBS for 30 min. Embedding was done on plastic molds and polymerization of OCT was achieved by incubation on dry ice, samples were then stored at − 80 °C. Sectioning of embryos was done with the help of cryotome; 7–10 μm thick cryo-sections were made and mounted on glass slides for microscopy.

## Additional files

**Additional file 1: Figure S1.** *efhc2*-Mo inhibits pre-mRNA splicing. (A) Schematic representation of zebrafish *efhc2* exon/intron organization, the target site of the splice-blocking *efhc2* morpholino (*efhc2*-Mo) and the forward and reverse primers used in RT-PCR for amplification of *efhc2*. (B) cDNA was prepared from embryos injected with *efhc2*-Mo and *efhc2*-MM, PCR amplified, cloned into pCR Blunt II Topo vector (Invitrogen) and sequenced using SP6 and T7 primers. Sequencing shows that injection of *efhc2*-Mo leads to deletion of exon-2 and exon-3 of *efhc2*.

**Additional file 2: Figure S2.** Effect of Efhc2 knock-down on nephron segmentation. (A) Morphological defects seen by injection of *efhc2*-ATG-Mo. Arrow indicates mild pericardial oedema. (B) WISH showing expression of *slc12a1* (DE), *slc12a3* (DL), *stc1* (CS) and *odf3* (MCC) in *efhc2*-ATG-Mo injected and standard control injected embryos. (C) Summary of defects caused by Efhc2 translation blocking morpholino.

**Additional file 3: Figure S3.** Efhc2 knock-down results in nephron segmentation defects. (A) WISH for pronephros segment or MCC specific markers on 24 and 48 hpf morpholino injected embryos. Expression of *slc20a1a* (PCT), *trpm7* (PST), *slc12a1* (DE), *slc12a3* (DL), *stc1* (CS) and *odf3* (MCC) in *efhc2* mismatch control (*efhc2*-MM) and splice-blocking morpholino (*efhc2*-Mo) injected embryos. (B) Summary of defects caused by Efhc2 knock-down.

**Additional file 4: Figure S4.** Phenotype caused by over-expression of *efhc2* mRNA. (A) *efhc2* mRNA injected embryos showed dose-dependent pronephros defects.

**Additional file 5: Figure S5.** Role of RA and Efhc2 in pronephros segmentation. Wild-type embryos or *efhc2*-Mo morphants were treated with DMSO, RA and DEAB. WISH showing expression of *slc12a1* (DE), *slc12a3* (DL), *stc1* (CS) and *odf3* (MCC). *efhc2*-Mo morphants treated with RA show expansion of expression domain of DE marker *slc12a1* and almost or complete loss of DL and CS markers *slc12a3* and *stc1* as compared with wild-type embryos treated with RA. The expression domain and the number of cells expressing MCC maker *odf3* was partially rescued by RA treatment in the morphants. DEAB treated *efhc2*-Mo morphants show expansion of DE, DL, and CS as compared with wild-type embryos treated with DEAB. The expression domain of *odf3* is reduced in DEAB treated WT embryos.

## Abbreviations

OCT: optimal cutting temperature compound
PBS: phosphate-buffered saline
Hpf: hours post-fertilization
