# Supplementary figures and images for: EF-hand domain containing 2 (Efhc2) is crucial for distal segmentation of pronephros in zebrafish
Source: Cell Biosci. 2018 Oct 16;8:53. doi: 10.1186/s13578-018-0253-z (PMC6192171; doi:10.1186/s13578-018-0253-z)

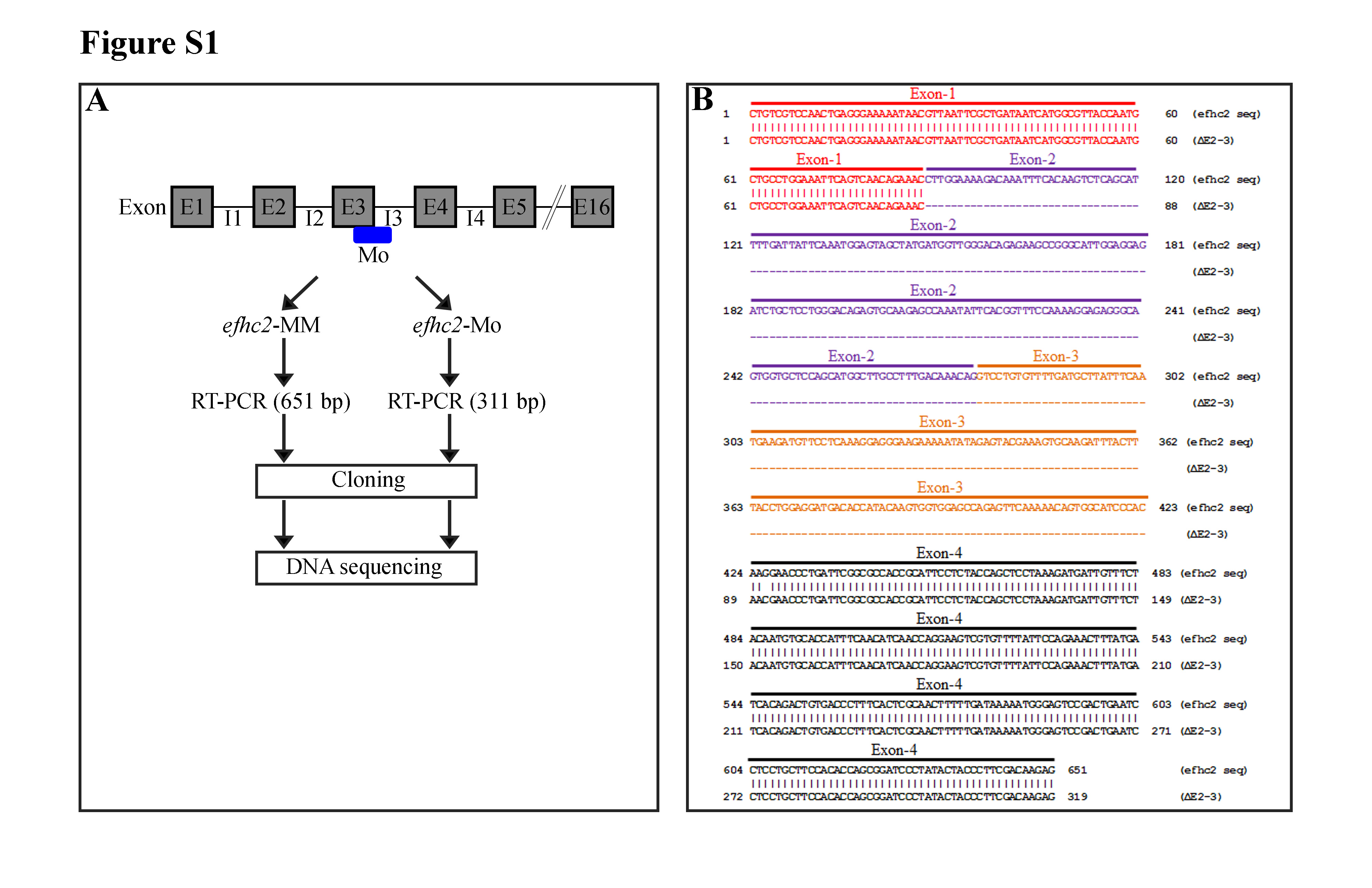

Supplement: Supplementary file 1 — Additional file 1: Figure S1. efhc2-Mo inhibits pre-mRNA splicing. (A) Schematic representation of zebrafish efhc2 exon/intron organization, the target site of the splice-blocking efhc2 morpholino (efhc2-Mo) and the forward and reverse primers used in RT-PCR for amplification of efhc2. (B) cDNA was prepared from embryos injected with efhc2-Mo and efhc2-MM, PCR amplified, cloned into pCR Blunt II Topo vector (Invitrogen) and sequenced using SP6 and T7 primers. Sequencing shows that injection of efhc2-Mo leads to deletion of exon-2 and exon-3 of efhc2. [file 13578_2018_253_MOESM1_ESM.jpg]

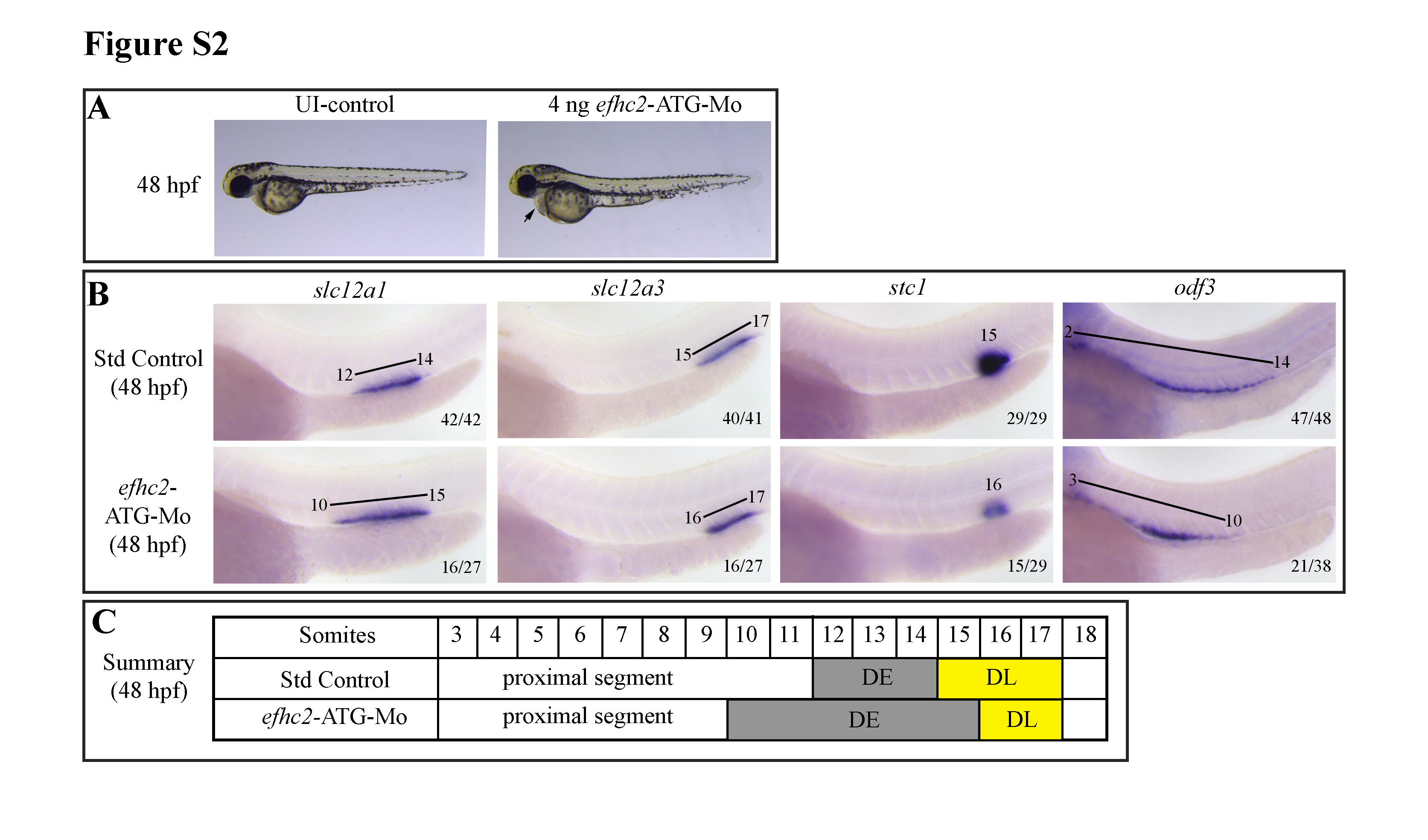

Supplement: Supplementary file 2 — Additional file 2: Figure S2. Effect of Efhc2 knock-down on nephron segmentation. (A) Morphological defects seen by injection of efhc2-ATG-Mo. Arrow indicates mild pericardial oedema. (B) WISH showing expression of slc12a1 (DE), slc12a3 (DL), stc1 (CS) and odf3 (MCC) in efhc2-ATG-Mo injected and standard control injected embryos. (C) Summary of defects caused by Efhc2 translation blocking morpholino. [file 13578_2018_253_MOESM2_ESM.jpg]

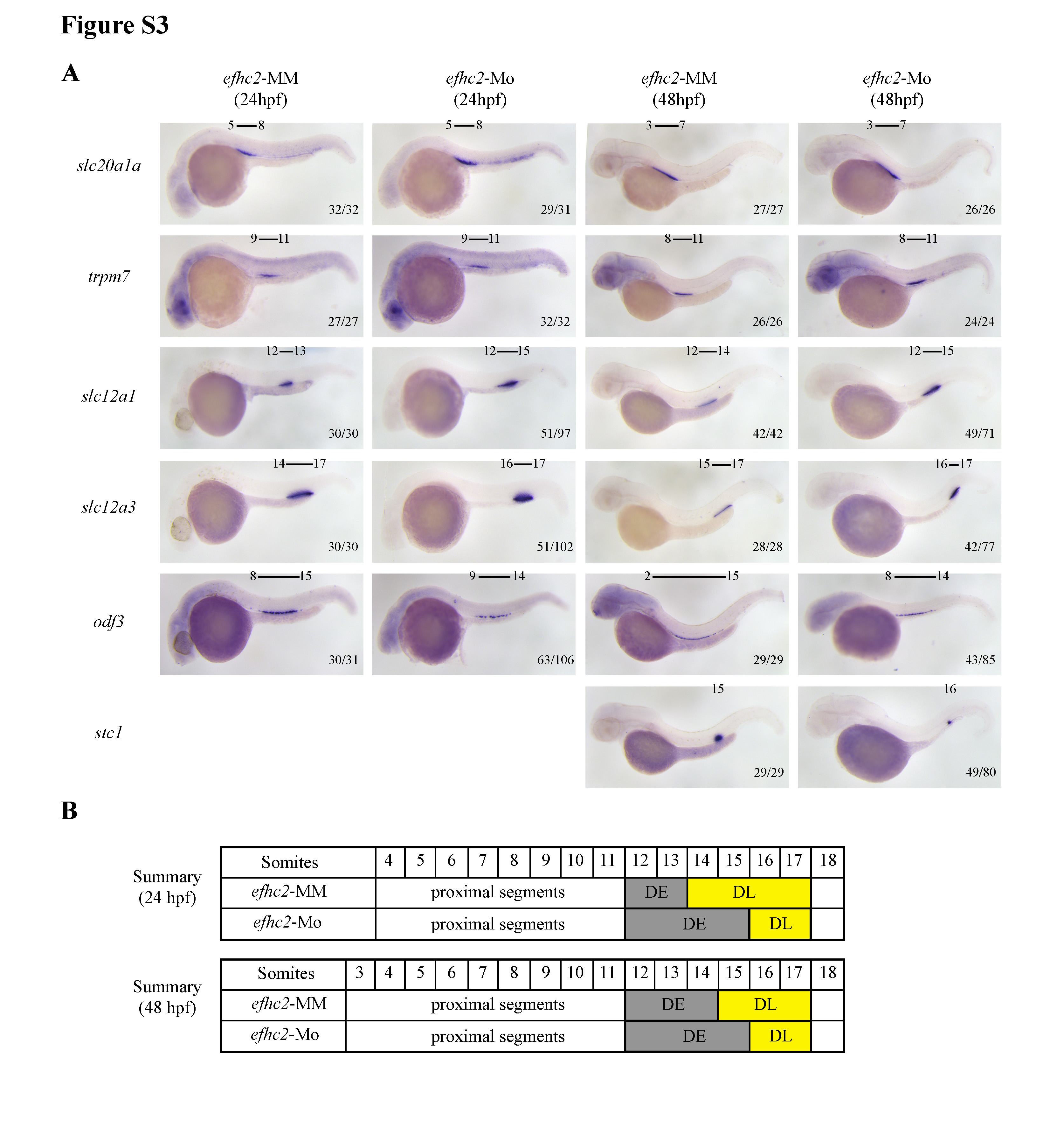

Supplement: Supplementary file 3 — Additional file 3: Figure S3. Efhc2 knock-down results in nephron segmentation defects. (A) WISH for pronephros segment or MCC specific markers on 24 and 48 hpf morpholino injected embryos. Expression of slc20a1a (PCT), trpm7 (PST), slc12a1 (DE), slc12a3 (DL), stc1 (CS) and odf3 (MCC) in efhc2 mismatch control (efhc2-MM) and splice-blocking morpholino (efhc2-Mo) injected embryos. (B) Summary of defects caused by Efhc2 knock-down. [file 13578_2018_253_MOESM3_ESM.jpg]

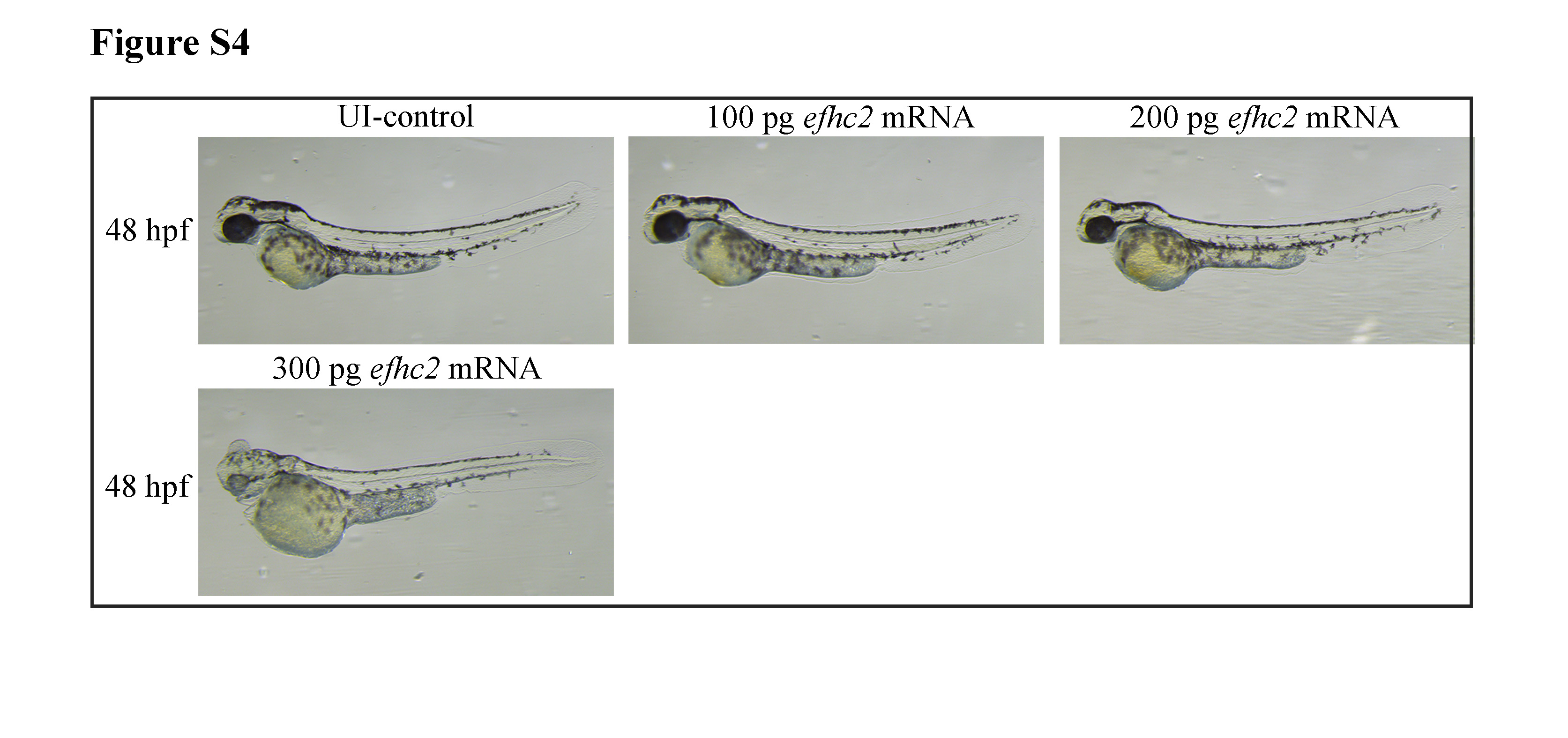

Supplement: Supplementary file 4 — Additional file 4: Figure S4. Phenotype caused by over-expression of efhc2 mRNA. (A) efhc2 mRNA injected embryos showed dose-dependent pronephros defects. [file 13578_2018_253_MOESM4_ESM.jpg]

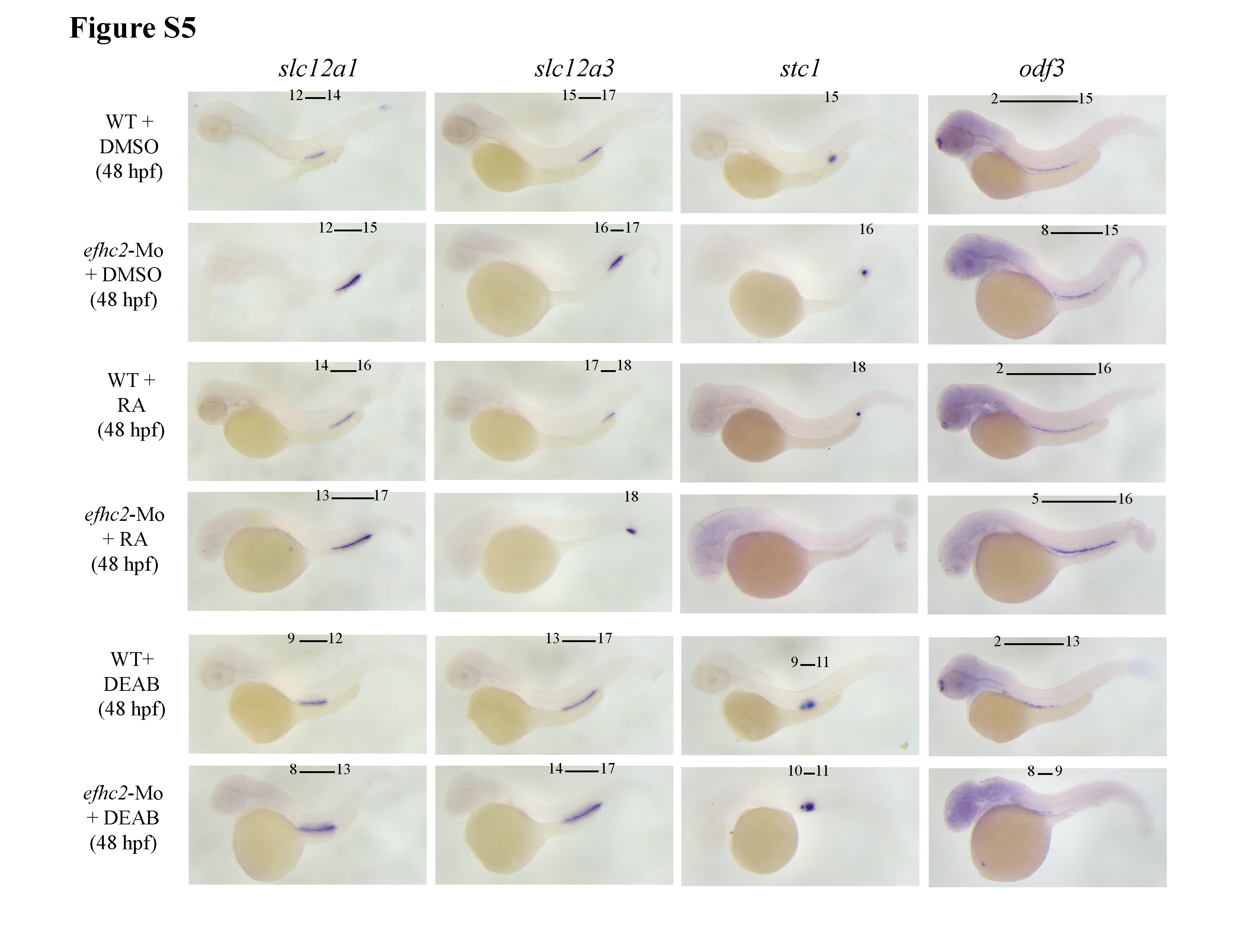

Supplement: Supplementary file 5 — Additional file 5: Figure S5. Role of RA and Efhc2 in pronephros segmentation. Wild-type embryos or efhc2-Mo morphants were treated with DMSO, RA and DEAB. WISH showing expression of slc12a1 (DE), slc12a3 (DL), stc1 (CS) and odf3 (MCC). efhc2-Mo morphants treated with RA show expansion of expression domain of DE marker slc12a1 and almost or complete loss of DL and CS markers slc12a3 and stc1 as compared with wild-type embryos treated with RA. The expression domain and the number of cells expressing MCC maker odf3 was partially rescued by RA treatment in the morphants. DEAB treated efhc2-Mo morphants show expansion of DE, DL, and CS as compared with wild-type embryos treated with DEAB. The expression domain of odf3 is reduced in DEAB treated WT embryos. [file 13578_2018_253_MOESM5_ESM.jpg]
